# Women’s collectives and social support: exploring pathways and impacts among forcibly displaced women

**DOI:** 10.3389/fsoc.2024.1409332

**Published:** 2024-09-11

**Authors:** Ilana Seff, Melissa Meinhart, Danielle Sarraf, Amna Abu Zuhair, Jacqueline Sofia, Racheal Atuheire, Jessica Lee, Nimo Ahmed, Catherine Poulton, Lindsay Stark

**Affiliations:** ^1^The Brown School of Social Work and Public Health, Washington University in St. Louis, St. Louis, MO, United States; ^2^Sitti Social Enterprise Ltd., New Hope, PA, United States; ^3^Transcultural Psychosocial Organization, Kampala, Uganda; ^4^Department of Global Health and Population, Harvard T.H. Chan School of Public Health, Boston, MA, United States; ^5^The Sisterhood Community Center, Yayasan Sisterhood Persaudaraan Perempuan, Jakarta, Indonesia; ^6^UNICEF, New York, NY, United States

**Keywords:** social support, gender-based violence, photovoice, women’s collectives, forcibly displaced populations

## Abstract

**Background:**

During humanitarian crises, women face both heightened vulnerabilities and a disruption to their social networks. Previous research points to the positive impact of women’s collectives in low-income settings, but less is known about the distinct forms, mechanisms, and consequences of the social support that emerges from these collectives, particularly for women affected by conflict. Recognizing the importance of social support in such contexts, this study utilizes the photovoice methodology to examine the dynamics of social support across women’s collectives in three distinct settings.

**Methods:**

This study recruited forcibly displaced women participating in women’s collectives in Indonesia (*n* = 5), Jordan (*n* = 12), and Uganda (*n* = 11). Photovoice, a participatory research method that centers the voices of study participants, was implemented across 7–8 sessions in each setting. Participants took photographs in response to four prompts and then discussed their pictures and lived experiences related to social support in facilitated discussion groups. Transcripts were analyzed using thematic analysis and a mixed inductive and deductive approach.

**Results:**

Across all study sites, participants highlighted the importance of five types of informal social support: emotional, instrumental, informational, companionship, and esteem support. Emotional support, one of the most prominently mentioned forms, served to buffer against stressors and enhance mental well-being. Instrumental and informational support facilitated meeting basic needs, accessing resources, and, in Uganda, improving members’ physical safety. Companionship support fostered a sense of belonging and shared identity, while esteem support validated members’ perspectives and aspirations and bolstered self-worth. In the Jordan collective, esteem support proved especially beneficial in countering the marginalization members experienced in their community.

**Discussion:**

Findings underscore the critical role of women’s collectives in providing diverse forms of social support to foster empowerment, agency, and resilience among forcibly displaced women. By centering the voices of participants and employing survivor-centered approaches like photovoice, this study amplifies the perspectives of women within these collectives, contributing to more inclusive and responsive humanitarian interventions. Future research should continue prioritizing women’s experiences and research questions, ensuring that interventions address their needs effectively.

## 1 Introduction

In addition to the direct and physical impact of conflict and natural disaster, humanitarian crises can have deleterious impacts on the intrinsic social fabric that exists between individuals, within families, and across society (Strang et al., 2020; Wachter and Gulbas, 2018). Women in humanitarian settings encounter more reductions in social networks and sources of support than men (Araya et al., 2007; Wachter and Gulbas, 2018), thus increasing their risk for outcomes associated with social strains. Moreover, gendered stressors and experiences, such as gender-based violence (GBV), have also been associated with social isolation and estrangement (Coker et al., 2002; Shumm et al., 2006). The evidence linking humanitarian crises with such risks highlights the importance of identifying programmatic solutions that center the lived realities of women affected by conflict and natural disasters. Building from the successes of group-based models in mental health and psychosocial programming in humanitarian settings (IOM, 2021; Silove, 2013), collective programming may answer this need by leveraging individual experiences and group dynamics to achieve shared goals. The establishment of what we refer to as “women’s collectives” are a result of decades of feminist action and ideology that unite women to wield the power to influence their lives, families, and communities.

Evidence from low-and middle-income countries (LMICs) has demonstrated the ability of collective programming for women and girls to have transformative impacts on personal agency and social capacities of participants (Brody et al., 2015). Identified as a notably cost-effective intervention (Cost-benefit analyses offend against the notion that life is priceless, 2019), women’s collectives in Ethiopia increased resilience among women (Horton and Morgan, 2017) and improved attitudes at the individual and community levels (Alemu et al., 2018). Specific to humanitarian contexts, women’s collectives have been shown to decrease domestic violence among women in Afghanistan (Schmeding, 2018), increase empowerment in Somalia (SomRep, 2018), and improve the health and wellbeing of GBV survivors in Kosovo (Medica, 2016). Further, a review of all forms of women’s collectives in LMICs indicated that reductions in intimate partner violence (IPV) were more likely to occur in conjunction with women’s increased decision-making power and stronger social connectivity (Chang et al., 2020). Still, there is a paucity of research examining *how* collective programming in humanitarian settings may have more expansive and sustained impacts on women and girls.

While limited, evidence has demonstrated that when women are enabled to come together to build social support, even if the initial program draw is not gender-specific, they are more likely to address issues for women (Buller et al., 2018; Chang et al., 2020). Social support, the perceived or actual exchange between individuals (Albrecht and Adelman, 1984; Cobb, 1976; Cutrona and Russell, 1990; Gottlieb, 1988; Jacobson, 1986; Kahn and Antonucci, 1980; Richmond, 1917), may provide a foundational component of women’s collectives that amplifies their impact. Social support may emerge through formal ties, such as service providers and/or organizations, or informal ties, such as those with peers and/or family members (Williams et al., 2004). Social support typologies are commonly identified as emotional, instrumental, informational, companionship, and validation support (Cohen and Lakey, 2000). Cohen et al. define emotional support as “confidant support, esteem support, reassurance of worth, attachment, intimacy,” instrumental support to include “tangible support, practical support, behavioral assistance, material aid,” informational support as “advice/guidance, appraisal support, cognitive guidance, problem solving,” companionship support as “belonging, socializing, [and] integration,” and validation (also referred to as esteem support) to include feedback and social comparison (p. 89) (2000). Ko et al. (2013) expand on this specific type of esteem support to include “the messages that help to promote one’s skills, abilities, and intrinsic value.” Cohen’s Stress-Buffering Hypothesis proposes that social support, particularly the perceived availability of social support, can protect individuals from the adverse effects of stress (Caplan, 1974; Cobb, 1976; Cohen et al., 2000; Cohen and Wills, 1985).

Building from the Stress-Buffering Hypothesis, Almedom created a foundational model applying social support within the framework of complex emergencies, asserting that “social support of the right type, provided at the right time and level, can mitigate the worst effects of war and displacement on victims/survivors (Almedom, 2004). This model asserts that emotional support, the verbal or physical exchange of empathy and care, is at the core of influencing the outcomes of war-induced mental health needs. However, given the increasingly protracted nature of conflicts, there is a need to understand how women’s collectives may give rise to other types of social support that then positively impact women experiencing forced displacement. One systematic review identified the unique influence of informal social support (i.e., peers, family, friends) among women survivors of violence in humanitarian settings (Meinhart et al., 2022). The review detailed that informal social support was particularly valuable at the neighborhood and community level, as well as within targeted groups such as peer networks of GBV survivors. When provided in a healthy and culturally appropriate way, social support can mitigate the psychological impact of stressors (Charuvastra and Cloitre, 2008; Cohen and Wills, 1985), like GBV, and enable healthy coping strategies (Cohen and Lakey, 2000). However, social support is highly contextualized, with personal, environmental, and cultural factors synergistically influencing the mechanisms, forms, and consequences of social support (Gottlieb and Bergen, 2010; Williams et al., 2004).

Although the literature recognizes that women’s collectives may provide a critical resource to achieving meaningful impact among and across members, there is limited evidence elucidating how social support emerges and serves as a mediator between women’s collective membership and improved mental health and well-being. This study recognizes that the pathways and efficacy of collective approaches to support women remain largely unexplored and the voices from women who lead and contribute to the activities of women’s collectives have yet to be amplified. Given the complexities associated with humanitarian programming and the novel nature of collective programming, we employed the photovoice methodology within three diverse collectives of women to understand the unique and shared ways that these collectives influenced members’ lives. This exploratory and descriptive study served to center participant voices and co-create answers to the research question: How do women’s collectives engender different types of social support and subsequent outcomes of improved well-being for forcibly displaced women?

## 2 Methods

Photovoice is a community-based participatory research method whereby participants capture, discuss, and analyze photos depicting their lived experiences in response to a series of focused prompts (Wang and Burris, 1997). Using photos as a medium to facilitate in-depth group discussion and qualitative data analysis skills, photovoice centers the voices of participants and promotes dialogue around their strengths, assets, and concerns. Further, participants are encouraged to collectively identify actions that can be taken to induce positive change in their communities (Catalani and Minkler, 2010; Lofton and Grant, 2021; Wang and Burris, 1997). As a qualitative research method with implicit skill-building components and discourse-based program implementation, photovoice shifts the power dynamics of traditional research studies and enables participants to “co-create” new knowledge alongside researchers (Castleden et al., 2008; Pearce et al., 2017).

In the present study, the photovoice methodology was employed to explore the emergence and role of informal social support in contributing to the well-being and safety of women’s collective members. While the research team was specifically interested in understanding the ways that women in the collectives shared and supported one another with regard to GBV, the photovoice methodology was used as a survivor-centered approach that did not include any questions explicitly mentioning GBV. Instead, we opened a space for participants to determine if and how to discuss violence or other lived experiences. While specific experiences of violence (sexual or any other form) were not discussed in great detail across study settings, the photovoice approach organically enabled conversations regarding the value of social support in relation to intimate partner violence, coping, and safety planning.

### 2.1 Recruitment and participants

Drawing on informal discussions with relevant stakeholders, we co-generated a definition of women’s collective as “a group of women in all their diversity who have organized and allied long enough to build robust social networks in their community” for the purpose of this study. This definition is purposefully open regarding composition and function to acknowledge the reality of complex, nuanced humanitarian contexts in which women’s collectives operate. The “collective” term reflects the centrality of women’s organizing within these groups. The terms “organization” or “group,” when applied in the humanitarian context, may overlook the ways that women collaborate–sometimes formalized, sometimes informalized, sometimes opportunistic, sometime strategic, and often building over time.

Eligible women’s collectives were required to operate within a humanitarian setting or be comprised of forcibly displaced women. No requirements on the initial purpose of the collective were included, nor were requirements regarding the top-down or ground-up establishment of collectives. An open call was made via UNICEF country offices in humanitarian settings, as well as within the study team members’ networks. Contacts at collectives who expressed interest in the study were contacted via e-mail. Collectives were purposefully selected to reflect a diversity in geographic location, participant characteristics, and type of emergency exposure. Three collectives agreed to participate, with one collective each in Indonesia, Jordan, and Uganda. An additional requirement of being 18-years of age or older was established for individual participants.

The Indonesian women’s collective, located in Jakarta, was founded in 2018 by refugee women who wanted to create a safe space for refugee women of all backgrounds and ethnicities. The collective offers vocational training, information on legal rights, literacy skills courses (English and Bahasa Indonesia) and, increasingly, includes advocacy on sexual and reproductive health and rights (SRHR) and (GBV). The collective also provides activities such as art, yoga, and health education and clinics. Fourteen women who were participating in a training workshop were invited to take part in the study. Five women from the collective, aged 21 to 47 years old and originating from Afghanistan, Iraq, and Pakistan, opted to participate in the study. Two women were not interested in; the other seven were ineligible due to age (*n* = 2), language (*n* = 2), or inability to commit to timeline (*n* = 3).

The women’s collective in Jordan comprises Palestinian refugees and is located in a camp outside of Amman. First created in 2010, the collective was formed to facilitate post-secondary educational opportunities through scholarships and financial assistance, encourage women’s meaningful participation in their local economic, social, and political systems, and decrease women’s isolation. A total of 12 women participating in a library volunteer program within the collective were invited to participate in the study, with all women agreeing to participate. Participants ranged in age from 19 to 34.

The women’s collective in Uganda was founded in 2017 for the purposes of generating group savings, providing physical assistance in times of crises, and helping harvest crops. The collective is located in a South Sudanese refugee camp and all members speak Acholi or Arabic. Eleven of the 12 collective members agreed to participate in the study (the 12th member served as one of the facilitators) and represented women ages 22–53.

### 2.2 Study procedures

Informed consent was collected from all participants prior to the program implementation via signature or thumbprint and included consent to have the reflection sessions recorded. A member of the research team presented the purpose of the study, the nature of participation, and measures taken to ensure confidentiality and privacy. Participants were also told they could withdraw from the study at any point. Participants were encouraged to ask questions about the research prior to providing consent and these discussions took place in Arabic, Acholi, or English, depending on participants’ language of choice.

The photovoice program took place over 7–8 sessions in each site. To mitigate power dynamics and issues around positionality of research participation, all Photovoice facilitators were affiliated with their respective collectives. The first session was used to explain the purpose of the study to participants and obtain informed consent, provide an overview of photovoice and each session, and share the prompts used to guide photo-taking. Importantly, the first session also emphasized the participatory nature of photovoice, highlighting that participants take on a researcher and participant role to ensure their voices and priorities are centered as part of the study. Each participant selected a pseudonym to be used throughout the program duration; participants’ real names were never spoken during discussions and were therefore not contained on audio files.

During the first session, participants were asked to take pictures before the second session in response to the following prompts:

1. What does being a part of [Women’s Collective] mean to you?
2. Can you share examples of a hard time in your life when you were helped by one or more members in [Women’s Collective]?
3. How do you feel when you are with members of [Women’s Collective] compared to when you are with other people?
4. What is the difference in the support you receive from members of [Women’s Collective] compared to the support you receive from others?

The discussion sessions (sessions 2 and 3 in Jordan and sessions 2–4 in Uganda and Indonesia) were used to facilitate group discussions about the pictures, prompts, and other themes related to the research questions. While photos guided initial discussions, facilitators were trained to probe as needed given the research topics of interest. Discussions took place with the full group in Indonesia and were carried out in English given the participants’ multiple countries of origin. In Jordan and Uganda, women were split into two groups for discussions; women were randomly divided in Jordan and divided by language in Uganda (one for primarily Acholi speakers and one for primarily Arabic speakers). Sessions were co-facilitated by a research team member and collective members pre-identified by the research team, who received training on the study and facilitation techniques. The study data comprised transcripts from these two to three discussion sessions across the three contexts.

Following these discussions, collective members participated in four analysis sessions to learn qualitative research skills, as well as co-create knowledge. These sessions included lessons and discussions on data analysis, transcript reading followed by coding practice, and discussions of common themes and overall findings. The program culminated in a final action planning session, where participants created final projects, including posters, to disseminate their main findings.

All information shared during the program was kept strictly confidential and data were deidentified. Password-protected audio files, which included only reference to participants’ chosen pseudonyms were shared with a select number of study team members. All study procedures were approved by the Health Media Lab’s Institutional Review Board (HML IRB Review #745INJU23).

### 2.3 Analysis

The reflection sessions in each site were audio-recorded, translated (when needed), and transcribed in English, by individuals who were present during the sessions and/or part of the study team. Given the sub-group and plenary discussions, a total of 14-transcripts were included in the final analysis. All identifying information was removed from the transcripts. Participants were referred to by their chosen pseudonyms, which are also used to label quotes in the Findings. An analysis team, comprised of six individuals (led by two lead authors), employed a mixed inductive and deductive approach to develop the codebook and a “flexible coding” process as per Deterding and Waters’ (2021).

Each analyst first read through one transcript and simultaneously drafted analytical memos. Team leaders then compiled common themes among the memos into a concept map, which was then transformed into a codebook. Throughout the creation of the concept map and codebook, the team continued to adjust the codes through transcript coding practice, codebook piloting/coding test sessions, and team meetings, until each team member felt that the final codebook sufficiently captured common and meaningful themes across the data. For example, once the codebook was formed, each analyst practice coded the same three transcripts, and then met to discuss any edits to the codebook, as well as code application discrepancies. Discrepancies in code application were resolved through consensus and refining the code definition until all analysts had a shared understanding. The 14 transcripts were divided among team members to code using the final version of the codebook. All transcripts were coded by two team members and any discrepancies in coding were resolved as a group.

Following coding, the team reviewed coded excerpts, identifying salient themes. Our primary research interests centered on the emergence and implication of social support in women’s collectives in humanitarian settings, whereby forms of informal social support were predominant. Findings for the present study are thus organized across forms of informal social support as defined by Cohen et al. (2000), as well as consequent outcomes of informal social support.

## 3 Results

### 3.1 Emotional support

Among all forms of social support discussed in the group sessions, emotional support remained one of the most salient and meaningful to the participants. The women frequently described how the collective helped guide them through difficult moments in their lives. For example, in the Ugandan collective, one participant shared how members provided her emotional support after the loss of one of her children: “The group members stood by my side, contributed some money, and also supported me a lot with their words” (Uganda). When explaining the difference between support received from within as compared to outside the collective, another participant reflected: “the support I receive from the women in this gathering … is like the food that the mother prepares for her children for nothing. Like her warmth, love and affection, where she does not wait for any rewards from her children, so when she feeds them, she gives them warmth, trust and love” (Jordan). For this participant, the collective’s altruistic and unconditional emotional support evoked affection, warmth, and trust.

While the initial purpose of each collective varied, women tended to emphasize the immense value-add of the informal emotional support gained through participating in the collective. One participant from the collective in Jordan expanded upon the impactful and meaningful emotional support she received from her collective: “One of the girls in this place supported me, even with a word or conversation. This is one of the things that is a big deal to me” (Jordan). When discussing the most critical modes of support from the collective, another participant similarly responded, “I felt that the most support we received as a group was psychological support. I mean, it was more psychological support and moral support than tangible support … the happiness, feelings, and inner peace that I mentioned to each of us, and the mutual support and acceptance of the opinions of others, and how this will create unity and interdependence between us” (Jordan). Even more than tangible support, this woman notes the importance of emotional support by way of mutual acceptance and respect in creating happiness and peace as well as fostering solidarity among the group. Another participant from the Jordanian collective echoed this sentiment, holding the emotional support she receives from the collective in higher regard than the formal trainings offered: “It is not necessary for me to come here because I want to do a workshop. Or work, no, it’s possible that a nice word or conversation at the collective makes me happy and gives me positive energy” (Jordan).

Participants also discussed the mental distress they experienced prior to joining the collective, crediting the emotional support from their fellow collective members as critical to ameliorating these feelings. As shared by a refugee woman in Indonesia: “all the refugees, you know, we all have stress … But my friend from [the collective] helped emotionally. So really this one is a big help” (Indonesia). Another participant from the Ugandan collective shared how members notice each other’s emotional and psychological needs and provide critical support, especially during times of extreme mental distress: “This photo represents GBV that was too much for me. I decided to commit suicide. As you see in the photo here, I was by the roadside squatting down, and immediately someone from the group approached and spoke to me” (Uganda). Previous mental distress also arose when one participant from the Ugandan collective reflected on her tumultuous past, where she “had no home” and “would move from one house to another,” never feeling “settled.” She noted that, once she joined the collective: “I was strengthened by the members and now I feel better in the group. Because of their constant support I now have a home” (Uganda). This participant attributed her newfound stability and emotional well-being to the supportive environment of the group, emphasizing the group’s role in providing a sense of belonging, stability, and hope that ultimately leads to the speaker’s improved well-being.

Moreover, participants directly linked the emotional support they received to various positive outcomes, including improved mental health, happiness, and hope. When describing her photograph of roses, one participant from the Jordanian collective articulated her gratitude for the collective:

Also, from the positive words they told us, words of motivation and phrases of psychological support, it is also possible that I feel grateful to them for the support they provide and the feelings they plant in me. I chose the picture of roses because I feel that the flowers express hope and optimism. They also give us solutions for problems. If you go to them with a problem, they will be able to help improve your psychology/mental health with just a few words or expressions, and it is a big support. (Jordan)

Similarly, a participant from the Ugandan collective acknowledged the transformative impact of a supportive group on her life, stating, “If I were not in the group right now, I would be mad, a thief, or even a prostitute. I am now upright mentally, because the group has helped me mentally and materially a lot” (Uganda). This participant portrays an improvement in her mental health, as she attributes her current mental well-being to the group’s influence, emphasizing the profound positive changes brought about by both emotional support and material assistance received through counseling. For her, the emotional support from her co-members serves not only to prevent potential adverse outcomes but also to foster a positive state of mental well-being.

Ultimately, in all three contexts, emotional support was especially salient for the participants, aligning with literature linking emotional support with aiding in enhancing self-esteem, reducing anxiety and depression, and motivating coping (Cohen et al., 2000). One participant from the Jordanian collective perfectly summed the juxtaposition of emotions while inside versus outside of the collective due to the emotional support received: “Feelings of joy, happiness, contentment, and comfort, giving, love, gratitude, appreciation, happiness, belonging, safety, pride, and love for the center or us. Our feelings when we are outside the center or collective: feelings of imprisonment, longing, lack of enthusiasm, loneliness, and depression” (Jordan).

### 3.2 Instrumental support

Across contexts, study participants consistently discussed the receipt and provision of instrumental support. While there were differences between the types of instrumental support provided across contexts, these discrepancies are likely at least partially due to differences in the initial purposes of the collectives and contextual variance. For example, given the focus of the Uganda collective on crop harvesting, only participants in the Uganda collective noted the importance of sharing physical labor for the purposes of farming: “As a group, we support each other with garden work, like planting different crops such as beans, maize, and some vegetables. Where a member needs something, like flour, charcoal, or salt soap, we support them as a group” (Uganda).

While direct financial provisions are paramount to conceptualizations of instrumental support, most mentions of financial provisions related to the formal support from and purpose of the collective, rather than informal support from fellow collective members. Instead, gift giving and collective intervention were important facets of indirect instrumental support. Collective members discussed the importance of gift giving across all three contexts. Gifted items were typically intended for children and included clothes donations for school and stuffed animals. For example, one participant from the Ugandan collective explained, “Sometimes they even give me their children’s old shoes and clothes” (Uganda).

Instrumental support and gift giving were especially useful for participants in times of stress. A participant from the Ugandan collective discussed how this material support also extended to her family members. As she discussed her photo of her paralyzed husband, she shared, “The group is still standing by my side to continue supporting him in life, and also to support my other family members, by visiting me and also giving me other physical support like sugar, soap and even some food items” (Uganda). A participant in the Jordanian collective described how the collective provided her with access to materials that she would not otherwise have, giving her the opportunity to succeed academically in college, where,

[A member of the collective] was always helping me, giving me English literature books to read, as well as books useful for me in my specialization and the same books related to my coursework in college. … I felt that I was walking this road, and she was always helping me giving me books and so on, and eventually I got there, and Alhumdallah, I got excellent grades in college (Jordan).

Materials were also exchanged to cope with the tough living conditions within the refugee camp. In Jordan, one participant highlighted how the collective provided instrumental assistance for the harsh winter: “I was a child, like 18, and it was winter … we could not afford to buy a heater for our home … So, someone from [the collective] at the time, they brought us a heater and gas, and I remember this till this day” (Jordan). This support left a lasting mark on Nada and her family, as she emphasized that even her mother “remembers this to this day.” When discussing the importance of material and physical support following loss and scarcity, one participant from the Ugandan collective described, “The second photo shows another hard time in life where I lost a loved one in death, my father, and that was a season of hunger, where there was no food, but every group member contributed whatever was available and came physically and supported me a lot” (Uganda). In Indonesia, one participant reflected on the provision of face masks during the COVID-19 pandemic. The collective member mentioned how this form of instrumental support was “very useful” and “really [a] necessity,” emphatically noting her gratitude, especially in a time of scarcity (Indonesia). In times of need, collective members came together across contexts to provide each other with material means of instrumental support to facilitate both practical problem solving and motivate coping (Cohen et al., 2000).

During difficult times, fellow collective members also contributed labor support. When one participant from the Ugandan collective drew comparisons between her experiences before and after joining the collective, she stated, “When I was not yet in a group, working alone was very hard. Now this first photo I took shows the women working as a group. When you are defeated with work, you can invite the members to support you. We also work together to support one another for free, and this helps us to pay for our children to go to school” (Uganda). This participant was able to receive direct support for herself and indirect support for her child by drawing on the solidarity of the collective to contribute, similar to gift giving.

Lastly, collective intervening to ameliorate interpersonal life stressors often arose as an integral form of instrumental support, particularly in Uganda, highlighting an important example of a non-tactile provision of instrumental social support. For example, one member of the Ugandan collective asked a fellow member for assistance when her husband prohibited her from attending the meetings: “I told her that if I were to ask my husband, he would not allow me or would even beat me more, so I requested Aling talk to him. She came one day to our home and tactfully talked to my husband, and he allowed me to go” (Uganda). As a result of her fellow collective member’s intervention, this participant was able to attend meetings without physical consequences. Another participant from the Ugandan collective shared how members of her collective intervened when she experienced intense mental distress after GBV: “I came to the collective and explained to them what I was going through, they comforted me and encouraged me with their words, they also went as a collective and talked to my husband where he listened and became better” (Uganda). While these findings align with formal definitions of instrumental support, they challenge implicit assumptions that instrumental support equates to tangible exchange.

### 3.3 Informational support

As was the case with instrumental support, forms of informational support available differed by site, likely due to both variations in the purpose of the collectives and local contexts. Importantly, informational support primarily arose in Uganda. Of the three contexts, the women in Uganda were more likely to discuss heightened life stressors around the inability to meet basic needs. Informational support may have been especially critical to support women in achieving the first tier of the hierarchy of needs and helping them realize physiological needs.

In the Ugandan and Indonesian contexts, informational support tended to help women achieve economic independence. For example, one participant from the Ugandan collective spoke to the importance of informational support in the formation of her business, which improved the quality of her life and ability to be self-sufficient: “they have also given me ideas for doing business, which has allowed me to not go to sleep hungry and to also buy some items for my family” (Uganda). Similarly, in Indonesia, one participant described how a fellow member encouraged her to join the [the collective] with information about the benefits of the collective. The participant recounted that her friend said to her, “I will help you to join the makeup classes and you will also get certificate” (Indonesia). By receiving information on how to access formal support offered by the Sisterhood, this participant was ultimately able to improve her livelihood.

Among the types of informational support listed by Cohen et al. (2000), advice was the most frequently mentioned in the data. One participant noted how collective members “share ideas and advise one another,” allowing the women to “solv[e] issues among themselves” (Uganda). In Uganda, participants often gave advice on coping with risks to safety, especially relating to GBV. One participant discussed how advice from the collective improved stressors related to GBV: “This photo shows a hard time in life where I was heartbroken due to the GBV I go through. When I am together with the women in the collective, I feel more settled and comforted because of their good advice than when I am not with them” (Uganda).

Importantly, advice given by collective members was often informed by relevant social and gender norms. This phenomenon can be observed in the following exchange, in which a facilitator, herself a member of the collective, offers advice to a participant whose husband is in prison:

Facilitator: What was the problem and the reason for taking your husband to prison, and how come you didn’t go to see him?

Participant (carrying infant): There is no proper reason for seeing him because he always beats me, whenever he is drunk, he comes home and beats me, almost every night. I didn’t feel like going there.

Facilitator: But still, you need to go and see him in the prison, because if someone does bad to you, you should forgive. (Uganda)

In this context, social norms around the acceptance and reporting of IPV also limited survivors’ options for responding to violence. When women did not feel safe reporting IPV to local authorities, advice and guidance from the collective served to help them contend with the “effects of GBV.” Responding to the facilitator, who inquired as to whether a participant had reported her abusive husband, a participant shared: “I sat on this issue for 4 years, and it intensified to the point that I started looking for advice from my fellow women … Concerning the local authority, the Sudanese culture does not permit a woman to report a man to any authority. For that reason, I did not. But the collective helped me and even [the collective] gave him some counselling” (Uganda).

Similarly, participants highlighted that advice played an especially crucial role when they faced dire situations. Sharing a transformative journey from profound isolation and mental distress to a newfound sense of resilience and mental stability, one participant from the Ugandan collective stated,

There has been a great difference for me between when I was alone and now, when I am part of the collective … Before I joined the group, I used to cry all the time and I was lonely, only planning to do bad things to myself like committing suicide, running away from family responsibility etc. With all the bad thoughts I was having, my group members were able to counsel me, and advised me to dig and plant crops, and take casual work in peoples’ gardens to get money to support myself. (Uganda)

For this participant, experiences of financial instability, loneliness, and interpersonal conflict converged to lead to severe mental distress and suicide ideation. However, the informational support alone allowed her to take action to improve her situation and ultimately become “strong in mind.” As such, advice from collective members frequently facilitated profoundly positive outcomes, often relieving stress and increasing comfort among participants. When one participant from Uganda reflected on a challenging period where she “was heartbroken due to the GBV,” she explained, “When I am together with the women in the collective, I feel more settled and comforted because of their good advice than when I am not with them” (Uganda). The advice and company from collective members provided a deep sense of security and solace for this participant. Another woman from the Ugandan collective described the strength and happiness derived from advice shared, noting, “when one is heartbroken, one gets strength from the visits and advice given by group members. This makes us strong and happy again” (Uganda).

### 3.4 Companionship support

Participants frequently expressed the importance of companionship support and belonging within their collectives in facilitating positive outcomes. Oftentimes, this sense of belonging was tied to collective solidarity, with women referencing their shared experiences and identities as enablers of belonging. For example, when referencing her previous exposure to GBV, one participant from the Ugandan collective felt comforted when she learned that others could relate to her experiences:

The photo here represents GBV happening, where the man chased my children out of the house, and they had to boil maize seeds outside because they were very hungry. I then decided to look for some comfort from fellow women. I also realized that I was not the only woman facing GBV, that many women go through that challenging situation, so we have to encourage one another. (Uganda)

Another participant from Uganda similarly expressed how she “realized that the problems that [she] was facing were similar to others” and that in all the stressful moments she was experiencing, including loss of family members, “the group stood firm with [her]” (Uganda). The impact of companionship ameliorated one participant’s stress levels: “Since I joined the group, I have realized that most of the problems we discuss and share here in the collective are similar. Through being part of this collective I have become stress free because I am able to share different ideas with women in the collective” (Uganda). By bringing women together to share and listen, the collectives provided an opportunity for the women’s experiences and feelings to be validated and normalized.

Even beyond contributing to a “positive affect,” as theorized by Cohen et al., companionship support emerging from the women’s collectives fostered a sense of safety and empowerment for members, so much so that one participant from the Jordanian collective considered the collective “as something sacred for us” (Jordan). More specifically, participants discussed the power of expressing themselves in a safe and trustworthy space and its transformative impact on their confidence and sense of self. For instance, two participants from the Indonesian collective expressed the power of shared perspectives within a support space:

Participant 1: Our viewpoints, we can share maybe in this room.

Participant 2: Our weak points. We can share, like, the pressure, which I feel like I have been stuck in a situation where I cannot decide my future. I cannot discuss it with my mom. I cannot discuss how I feel, how pressure I feel from outside and also from inside, but I can discuss it with them. And usually I discuss about our cases and process and the stress which some of the ladies already experienced and get rid of them. Since I joined, I have discussed with them. (Indonesia)

Furthermore, members felt that convening with women, who held shared gendered experiences, was critical to fostering their sense of belonging. In Jordan, one participant discussed the realities of her community, noting the lower status of women and girls in the refugee camp relative to men and boys. This reflection then prompted her to add,

Because these are girls who live in the same place with the same circumstances, they may understand me, whereas if they are a brother or a father, it is a difficult thing to express your feelings and tell them, because males have a hard time with feelings, not like females. I mean, I understand Qamar, what is wrong with her, because I am going through such things while I am talking to them. I have feelings like this.

One participant from the Indonesian collective distinguished between the special support that can be offered by women versus men. When answering a question about the impact of the gender of a supporter on one’s sense of comfort and safety, she says,

..We have some problems emotionally especially if we [are] receiving help from the men, we are not comfortable like [with] women. This is very different, men and women. Also sometimes we have for example here we learning right? We are always woman. Sometimes we discuss our personal problem also. If we have any issue, we discuss with the woman also. But outside we cannot, right? But also we trust each other. That's why many times we discussed our personal matter also but outside with men we cannot discuss. It's really a big difference for me. (Indonesia)

This participant thus compares support received from men, often associated with emotional discomfort, with the ease and trust she feels when sharing personal matters with women. This quote illuminates how comfort and trust can emerge in a dedicated supportive environment and the impact of gender dynamics in shaping the participant’s sense of belonging and openness. She later continued to highlight how support from women contributes to feelings of solidarity, as she explained the meaning of a sisterhood to her: “First by sisterhood [we] mean to support and [receive] assistance from women. Especially from women. Here highlighting the point and solidarity of women in their shared experience” (Indonesia). For many participants, there was value in sharing like experiences and emotions in a designated space for women.

### 3.5 Validation

Validation, or esteem support, was also a salient form of social support within the data. This form of social support was most prominently featured in the Jordan study site. As the women participants in Uganda and Indonesia likely experience constant and severe life stressors more frequently than those in Jordan, informational and emotional support may hold higher value in these contexts where physiological needs must be prioritized. Alternatively, in Jordan, esteem support may have been more pertinent given those survival needs have already been met. Participants from the collective in Jordan discussed their appreciation for esteem support received within the backdrop of their local context, often noting the constraints on their ability to express opinions outside of the collective. One woman discussed her perception of gender inequality within her community, where only men and boys have freedom and a voice:

I mean, in our society, boys have more freedom and have more authority than sisters. This keeps things stagnant. … and it’s limited. It’s shutting down all of our ideas. For all our ambitions, for example, I wish that I am a boy who can do what I really want. They go out whenever they want, they go wherever they want, they sleep whenever they want, they travel. (Jordan)

Another participant outlined the disenfranchisement she feels outside of the collective, where “it is possible that the people outside the collective will not listen to you at all. They may destroy us as well. In the surrounding society they tell you that you cannot, you will not achieve, you will not achieve.” (Jordan).

Thus, having a voice and feeling heard were two especially important aspects of esteem support, both of which facilitated feelings of empowerment. One participant outlined the validation she felt when collective members listened to her point of view and ‘saw’ her: “It is possible that in your normal life you will not find anyone who listens to you, but here you will not find anyone who does not listen to you, hears your point of view, sees it, supports you, and may come and listen to inspiring stories that encourage them to come here” (Jordan). Using her photo of birds, another participant described the empowering feeling of “unleashing oneself” once she was able to express her thoughts and opinions: “this is a photo of birds, I liked taking this photo, it means unleashing oneself, I mean how we unleashed ourselves, and when I am in the gathering, I feel that I can describe what’s inside me. I feel like unleashing oneself exists among birds and us” (Jordan). Similarly, another woman described the strength and empowerment she felt as a result of her ability to share without fear of criticism:

In this community I feel strong, I feel that I could express my opinion with others, I mean I can positively share my feelings and thoughts, and don’t receive negative criticism, I mean they won’t criticize me for me. So, I feel strong here like that … What does sunrise mean, it means a new beginning, happiness, I mean seriously when I am with them, I feel happy … I feel that I can have an impact in the community and the community is impacting me, and not like someone who is there not doing anything. (Jordan)

As demonstrated in this quote, participants felt that their sense of empowerment not only had a direct impact on themselves, but also an indirect impact on their greater community. This theme also carried over to Indonesia, where one participant highlighted the value of open and non-judgmental discussions in a shared space. She expressed that “when we are with women in sisterhood [we] can find empowerment through collective strength, inspiring each other to pursue [our] goals and dreams” (Indonesia). Empowerment through collective strength in the sisterhood illustrates how mutual inspiration within a supportive community encourages individuals to pursue their aspirations, reinforcing the idea that each voice matters. Therefore, these simple acts of collective listening, acknowledging, and making space for co-members’ feelings and thoughts were often enough to make the women feel supported and empowered.

Esteem support also bolstered self-confidence, enhancing participants’ feelings of their intrinsic value. One participant equated her experience to a growing plant that she portrayed in her photograph. She discussed the emergence of her self-confidence and its interplay with belonging as a result of collective membership, when she said, “My feeling in this place comes out like this is a small seed, so they planted me in this place and I grew. I became more ambitious. My self-confidence has increased, and I have a sense of belonging to the place I am in” (Jordan).

Participants also directly associated esteem support with skill-building. For example, one woman described how the collective encouraged self-acceptance and boosted her self-confidence through a propagation of new abilities, including computer skills. She described, “They raised my morale more and made me feel that I was proud of certain abilities. … They were encouraging and strengthened my self-confidence. I felt self-confident from acquiring new skills” (Jordan). Esteem support in many cases was therefore inextricably linked to improved self-esteem itself. When describing her photograph of a speaker in an assembly, the same participant articulated the collective’s ability to uplift participants, which helped build empowerment and self-confidence.

They taught me that and encouraged me more. They raised my morale more and made me feel that I was proud of certain abilities. On the contrary, they taught me and encouraged me and did not show me that I was less or that I did not have abilities or that I did not have skills. They were encouraging and strengthened my self-confidence. (Jordan)

As demonstrated in this quote, feelings of pride, encouragement, and strength were all important outcomes of esteem support.

Esteem support also worked to improve feelings of mental distress and hopelessness by increasing members’ sense of intrinsic value and agency. One participant shared that outside of the collective she “feel[s] alone, sad, no hope, no effort, not able to share with anyone, so I feel like I am not ok,” while within the collective, “they encourage you, that you have these capabilities, that you could do something of yourself” (Jordan). A participant from the Jordanian collective used a “flower branch” on an “old wall” as imagery to discuss the feelings of hope, positivity, and productivity that resulted simply from having a place to express her thoughts: “And when I come to the center, I feel hope, hopefulness, and positive energy, and when I talk with the girls I find my private space, where I could share my thoughts, and they share their thought, I feel that I am a productive person within the community, and even if my impact is little like a butterfly, it’s a positive impact” (Jordan).

## 4 Discussion

Across all three women’s collectives included in this study, members discussed benefiting from five types of informal social support: emotional, instrumental, informational, companionship, and esteem support. While each type of social support manifested in multiple ways, clear patterns emerged across the three sites. Emotional support, one of the most prominent types of support mentioned, often presented in the form of sympathetic and encouraging words and consistently served to buffer against stressors and improve the mental health of women across contexts. Instrumental and informational support, in contrast, helped women to meet their and their children’s basic needs through the receipt of in-kind support and suggestions related to income-generating activities, respectively. Companionship support was frequently operationalized as promoting a sense of belonging through shared experiences and identities—particularly as they relate to womanhood—and esteem support was typically observed as validating members’ feelings, ideas, and value; both types of support contributed to feelings of empowerment, agency, and purpose for participants. Unique features of social support were also observed between collectives, alluding to the criticality of context-specific considerations when working with women’s collectives. In Uganda, instrumental support in the form of intervention also emerged as a key resource for women experiencing controlling behaviors or IPV at the hands of their partners; in Jordan, validation of members’ opinions and goals helped to mitigate impacts of the gender inequitable norms germane to this setting. Overall, findings indicated that the unique and shared presentations of social support were vital in improving the lives of women participating in the collectives.

The importance of building social support among women in humanitarian settings and throughout the forced migratory process is particularly critical when considering that women experience diminished social support in these contexts (Wachter and Gulbas, 2018b). Social support, and emotional support in particular, have been shown to be at the core of influencing the outcomes of war-induced mental health needs (Almedom, 2004), which supports global evidence indicating that social support can protect individuals from the harmful effects of life stressors (Cohen and Lakey, 2000). Findings from this study expand on the value of social support, highlighting its utility in mitigating other sources of distress for forcibly displaced women, including difficulty meeting basic needs and concerns around emotional and physical safety. Complex emergencies can also exacerbate existing gender inequitable norms, limit women’s mobility and censor freedom of expression (Lafrenière et al., 2019; Trapped Populations|18|Limits on mobility at times of crisis|R, 2014). Across all three collectives, despite their unique contexts and initial reasons for conception, we found that social support at least partially mitigated impacts of all these concerns, in turn nurturing women’s sense of belonging, self-worth, hope, agency, and mental well-being.

Findings also demonstrate positive impacts of women’s collectives – regardless of the collective’s initial objective in coming together – for women at risk of or who have experienced GBV. Given the increased risk of GBV in humanitarian emergencies, understanding the mechanisms through which women’s collectives may innately support survivors is critical. Previous research among GBV survivors in humanitarian settings indicates that social support can enable help-seeking behavior and emotional recovery (Stark et al., 2021). In this study’s Uganda site, instrumental support in the form of direct intervention from other members of the collective proved crucial for intimate partner violence survivors, with one participant sharing that direct intervention by collective members led to the cessation of her abuse. While other forms of social support may not necessarily prevent future instances of violence, they can help to minimize negative consequences for survivors. Our findings suggest that the combination of emotional and companionship support may give rise to a dynamic similar to that of a more traditional “support group.” Flasch, Murray and Crowe found that connecting with and receiving support from other survivors was critical for overcoming abuse and achieving mental wellness (Flasch et al., 2017).

However, it is important to note that the social support collective members can provide survivors is influenced by several contextual factors, including social norms around IPV, mobility constraints, and broader safety concerns associated with displacement. In some cases, support that ultimately improves a woman’s mental well-being may not also improve her physical safety, as was the case when a woman in the Ugandan collective felt more at peace after being encouraged to remain with and accept an abusive partner. Additionally, future research should explore the conditions in which a woman participating in women’s collectives serves as a trigger for IPV. While the members of the collective in Uganda were able to successfully deescalate a situation of IPV against one of their fellow members, the survivor’s desire to participate in the collective was itself the precipitant of the abuse. Women’s collectives, with all their intrinsic value, may pose their own risks for members and may not necessarily take the place of stronger mental health supports and programming.

In the present study, two forms of social support—esteem and companionship support—were often discussed by participants in ways that were inextricably linked to solidarity and shared experiences as women. The Intersectional Theory of Cultural Repertoires in Health (RiH) helps to shed light on these dynamics, highlighting the value in drawing on the dimensions of one’s identity that best enable access to resources for effective coping (Bennouna et al., 2022; Seff et al., 2024). RiH puts forth that all individuals have access to cultural repertoires based on their membership in multiple and sometimes overlapping groups. Cultural resources that strengthen one’s sense of belonging to a particular group—such as solidarity, safe spaces, and collective action—can serve as sources of emotion-focused coping through provision of esteem and companionship support, while at the same time engendering meaning-focused coping (Park and Folkman, 1997). Through these collectives, participating members were able to identify ways in which their experiences and identities overlapped with other women in their community, consequently fostering a sense of purpose and belonging. In the Jordanian collective, the gender inequitable community in which participants lived, the shared experiences of marginalization that members faced, and the opportunity to come together through the collective all converged to enable discussions around voice that stemmed from an implicit understanding between members. Women participants found in the collective a space to share their opinions and aspirations, an opportunity they juxtaposed with the explicit muting of their voices in their community. Further, beyond serving as a safe space to share thoughts and feelings, the collective and its members actively affirmed each other’s perspectives and “intrinsic worth” through esteem support, giving some women a sense of purpose for the first time (Kabeer, 2005). Importantly, however, few examples were encountered whereby validation within the Jordanian collective translated to challenging inequitable gender norms within members’ household spheres. Studies from other contexts have similarly found that less accepting attitudes toward GBV among women’s collective members were not found among their partners (Alemu et al., 2018; Horton and Morgan, 2017), pointing to a need to examine how changes in attitudes among women may or may not influence norms and to what extent programming can address social and gender norms at a household or community level.

As was observed with the collective in Indonesia, where members originated from multiple countries, traditionally prioritized dimensions of identity—such as age, ethnicity, and others—need not necessarily be the factors that bind women together. Considering intersectional theory and RiH, findings demonstrate that diversity of membership can enable a broader sense of sisterhood beyond traditional bounds and may enable women to express experiences or beliefs that would be seen as taboo within their communities (Bennouna et al., 2022; Crenshaw, 1991). For example, an evaluation of a community-based PTSD intervention for survivors of intimate partner violence, for example, found that respondents valued their shared identity as survivors, beyond any other identity dimensions (Kelly and Pich, 2014). Further, identifying one’s experiences and identity in others can be its own form of peer support (Mead et al., 2001). Fostering solidarity through these shared experiences and resonant dimensions of identity offers promise for improving women’s mental health in complex emergency settings.

This study has several limitations. Most of the co-authors and data analysis team members are white, highly educated women living in the United States, which may have impacted interpretation of certain participant quotes. To mitigate the impact of this bias, analysis was carried out as a collaborative process and transcripts were reviewed by in-country teams for accuracy. The conceptual mapping process, which informed the initial code creation, was informed by summative reporting by in-country teams who identified emergent themes from the photovoice discussions. Preliminary findings were also shared with each in-country team who, in turn, were encouraged to communicate findings with the women who participated in the study. The recruitment of women’s collectives for participation in this study resulted in the identification of three diverse collectives, with respect to both contexts and participant characteristics. While this diversity was helpful in capturing the scope of support that may emerge in collectives, it presented challenges in finding structural throughlines across settings. Nonetheless, several types of social support were found in multiple study sites, pointing to the value of women’s collectives regardless of their origin or attributes. The consistency of findings between these diverse collectives also encourages the likelihood that these findings reflect similar facets of other collectives. While photovoice sessions for the Indonesian collective were conducted in English to accommodate site-specific preferences, English was not the native language of participants. As a result, some quotes in this context may not have been correctly understood or interpreted as intended by the study team. Similarly, while sub-groups discussions were specific to primary language, plenary discussions were held in a shared language; thus, limiting the interpretability of plenary quotes. Finally, while our definition of women’s collective provides a useful guide, it is not meant to be prescriptive as we cannot truly know the composition or exact function of all women’s collectives, particularly in the complex and nuanced reality of humanitarian settings.

## 5 Conclusion

This study sought to explore how women’s collectives give rise to different forms of social support that in turn serve to bolster the mental health and well-being of forcibly displaced women. Through the use of the photovoice methodology, members of women’s collectives served as active participants in generating learnings around these pathways. Across three diverse study sites, examples of emotional, instrumental, informational, companionship, and esteem support emerged as critical mechanisms for fostering empowerment, agency, and mental well-being among participants. While women viewed their collectives as spaces to give and receive emotional support, they also discussed how instrumental and informational support emerged as pathways to meeting their basic needs. Further, findings revealed that the collectives offered members the opportunity to identify shared identity dimensions and experiences, which in turn promoted solidarity and a sense of belonging. Future research might usefully explore what conditions must be in place to ensure full emergence of these forms of social support as well as how women’s collectives can safely and effectively translate increased empowerment and agency into action. Importantly, future research on the benefits of women’s collectives should further center the voices of women themselves, prioritizing their research questions and experiences to ensure that interventions are meaningful and effective. By amplifying the perspectives of women within these collectives, future research can contribute to the development of more inclusive and responsive approaches to promoting gender equality, well-being, and resilience in humanitarian contexts.

## Data Availability

The raw data supporting the conclusions of this article will be made available by the authors, without undue reservation.
